# Business Versus Ethics? Thoughts on the Future of Business Ethics

**DOI:** 10.1007/s10551-022-05241-8

**Published:** 2022-10-04

**Authors:** M. Tina Dacin, Jeffrey S. Harrison, David Hess, Sheila Killian, Julia Roloff

**Affiliations:** 1 Smith School of Business, Queen’s University, Kingston, ON Canada; 2 Robins School of Business, University of Richmond, Richmond, VA USA; 3 Ross School of Business, University of Michigan, Ann Arbor, MI USA; 4 Kemmy Business School, University of Limerick, Limerick, Ireland; 5 Department of Management and Organisation, Rennes School of Business, Rennes, France

**Keywords:** Corporate purpose, Human rights, Strategy, Stakeholders, Unethical business models, Culture, Social enterprise, Future of business ethics

## Abstract

To commemorate 40 years since the founding of the Journal of Business Ethics, the editors in chief of the journal have invited the editors to provide commentaries on the future of business ethics. This essay comprises a selection of commentaries aimed at creating dialogue around the theme *Business versus Ethics?* (inspired by the title of the commentary by Jeffrey Harrison). The authors of these commentaries seek to transcend the age-old separation fallacy (Freeman in Bus Ethics Q 4(4):409–421, 1994) that juxtaposes business and ethics/society, posing a forced choice or trade off. Providing a contemporary take on the classical question “if it’s legal is it ethical?”, David Hess explores the role of the law in promoting or hindering stakeholder-oriented purpose and governance structure. Jeffrey Harrison encourages scholars to move beyond the presupposition that businesses are either strategic or ethical and explore important questions at the intersection of strategy and ethics. The proposition that business models might be inherently ethical or inherently unethical in their design is developed by Sheila Killian, who examines business systems, their morality, and who they serve. However, the conundrum that entrepreneurs are either lauded for their self-belief and risk-taking, or loathed for their self-belief and risk-taking, is discussed by M. Tina Dacin and Julia Roloff using the metaphor of taboos and totems. These commentaries seek to explore positions that advocate multiplicity and tensions in which business ethics is not either/or but both.

## The Intersection of Law and Business Ethics

**David Hess**

## Introduction

The relationship between law and business ethics has been a core research area across the 40 years of the Journal of Business Ethics. Indeed, the very first issue of the journal asked the question of whether legally allowed bluffing in labor negotiations was ethical (Carson et al., 1982). More recent research has flipped that question of “if it’s legal, is it ethical?”, and used Uber’s rideshare expansion strategy—which involved directly violating laws regulating taxicab services—to ask if illegal actions are always unethical (Young, 2019). These are two simple illustrations of the numerous ways that law, public policy, and business ethics intersect.

The Journal of Business Ethics encourages researchers to consider how business ethics scholarship informs the goals of the laws that regulate business and our understanding of how to improve individual and organizational compliance with those laws. Due to the constant changes in society, the questions on the role of the law in encouraging ethical business behavior are continuously evolving. For example, technology advancements raise questions of whether and how to update laws that protect the privacy rights of employees and consumers. Likewise, the use of artificial intelligence in employment decisions and the operation of autonomous vehicles raises important issues related to employment law and products liability law, respectively. Societal changes leading to an increased attention to issues of diversity, equity, and inclusion, raises issues of the role of the law in increasing the numbers of those that identify as women, underrepresented minorities, or LBGTQ+, in management and corporate boardrooms.

### Corporate Purpose, Environmental and Social Governance, and Business and Human Rights

The purpose of the corporation, which has been an important research issue for the journal, is another key area where law, public policy, and business ethics have intersected. Recently, the debate over corporate purpose has reached a new level of public attention due to the Business Roundtable—an organization made up of CEOs of leading corporations in a wide range of industries—revising its prior statement on corporate purpose to shift from a shareholder maximization view to the adoption of a stakeholder view (Loughran et al., 2022). Although this is an important development, skeptics question CEOs’ commitments to the Business Roundtable’s statement. This raises issues of how the law can be structured to hold corporations legally accountable for such commitments. For example, there are questions of whether legislators should adopt a new type of corporation—such as the benefit corporation—as an alternative to the existing shareholder value focused corporation. Likewise, business ethics scholars have considered changes to corporate governance, including stakeholder representatives on boards of directors and altering directors’ fiduciary duties.

Coinciding with the Business Roundtable’s statement, environmental, social and governance (ESG) issues have become a top priority for corporate boards. One survey found ESG was the top issue that shareholders sought to discuss with the board, ahead of both executive compensation and strategy oversight issues (PwC, 2021). As shareholders demand more and better information on corporations’ performance on sustainability matters, there are questions of whether there should be mandatory corporate disclosures on ESG matters. The European Union’s Corporate Sustainability Reporting Directive (CSRD) shows a movement away from voluntary reporting and toward compliance with mandatory standards. As those standards are disclosed and implemented, business ethics scholars must help guide such legislation by examining its effectiveness in changing corporate behavior and identifying necessary reforms. As seen from the existing work on nonfinancial disclosure in the Journal of Business Ethics, this legislative evaluation effort will benefit from the wide range of disciplinary perspectives represented by business ethics scholars.

One key aspect of the “social” factor in ESG is respecting human rights. The field of business and human rights (BHR) did not exist when the Journal of Business Ethics was founded in 1982. This field developed primarily with legal scholars and focused on mandatory obligations and holding corporations accountable for the harm they caused, as opposed to a corporate social responsibility perspective focused on business making voluntary, positive contributions to human rights (Ramasastry, 2015). Although there may be a gap between the fields of BHR and CSR, there are opportunities for connection (Ramasastry, 2015), especially for research related to the journal’s section on law, public policy, and business ethics.

In the last decade, BHR has rapidly moved from soft law to hard law. The 2011 United Nations Guiding Principles on Business and Human Rights (UNGPs) solidified support for business having a responsibility to respect human rights. This support—and perceived lack of corporate commitment to human rights—led to the adoption of legislation on BHR issues in various countries and the formation of a United Nations working group to develop a binding treaty. Included within this discussion of mandatory human rights obligations are considerations of how a “smart mix” of soft law and hard law mechanisms can help “reach beyond the limits of conventional public law” (Buhmann, 2016, p. 710). In addition to regulatory actions, courts in some jurisdictions are starting to show a willingness to hold corporations liable for the acts of their subsidiaries or supply chain partners in foreign countries, which may further impact business practice (even when the litigation is unsuccessful) (Schrempf-Stirling & Wettstein, 2017). As the legal environment surrounding BHR continues to evolve, business ethics scholars can provide valuable guidance on the way forward. The remainder of this commentary will provide a brief overview of recent developments in the law on BHR and provide some illustrations where scholars at the crossroads of law, public policy, and business ethics have provided valuable contributions, which also shows the way for future contributions in this area.

### Business Ethics and Business and Human Rights Legislation

Governments have approached BHR legislation in a variety of ways. The scope of the laws either focus on human rights generally or are targeted toward specific issues, such as conflict minerals, modern slavery, or child labor. There are also differences in obligations and potential liabilities; a law may be limited to disclosure requirements, may mandate the undertaking of human rights due diligence (HRDD)—which is a process for identifying, preventing, mitigating, and remediating negative human rights impacts—or may provide for legal liability to victims of human rights abuse. For example, targeted, disclosure only laws include the UK Modern Slavery Act and the California Transparency in Supply Chains Act (CTSCA). These laws only require corporations to disclose what actions, if any, they have undertaken to ensure that modern slavery is not present in their supply chains. The Netherlands’ Child Labor Due Diligence Act, on the other hand, requires corporations to conduct due diligence to determine whether there is a reasonable suspicion that child labor was used in the production of the company’s goods and services. If such a finding is made, then the corporation must develop a plan of action to address the issue. France’s Duty of Vigilance law mandates HRDD, disclosure of the vigilance plan, and opens the company up to potential civil liability if inadequate HRDD results in a human rights violation.

Business ethics scholars have already started to examine these laws and propose reforms. The few examples of scholarship discussed here provide inspiration for future research possibilities. For targeted disclosure laws, Birkey et al. (2018) examined corporations’ public reports under the CTSCA and found that companies comply with the law through mostly symbolic disclosures. Interestingly, although there is much discussion of investors pushing for increased disclosure of ESG information, this study raises the question of whether symbolic compliance is due, in part, to the fact that many investors “interpret increased disclosure as potentially costly in terms of firm value” (Birkey et al., 2018, p. 837).

Beyond market participants, a disclosure regime relies on non-market actors, such as NGOs, to utilize disclosed information to influence change in corporate behavior. Thus, Islam and Van Staden (2022) analyzed the effectiveness of modern slavery disclosure laws by interviewing key stakeholders, including anti-slavery NGOs. This research provides insights into the tensions between the ability of such laws to work toward long-term change and their limitations due to regulatory capture by interest groups focused primarily on reducing business risk. In addition, this study, along with others such as Pinnington et al. (2022), use normativity theory to provide insights into the perceived legitimacy of these disclosure-based regulatory regimes, which calls for additional research into how to enhance their legitimacy. Pinnington et al. (2022) also open new possible avenues for improving disclosure regimes by examining corporations’ disclosures on their discovery efforts, including corporations’ discussion of the “known unknowns” in their supply chains and how they plan to fill that information gap. Business ethics scholars are also well-positioned to explore how government can improve the regime through “guidance, leadership, training and scrutiny” and not simply the use of coercive power (Pinnington et al., 2022). For example, public procurement practices, which focus on the government providing positive incentives, as opposed to using coercive power, are a potentially valuable tool in need of further research (Martin-Ortega, 2018).

Turning to HRDD practices, research has examined how firms respond to external pressures, such as regulatory pressures. For example, in the area of conflict minerals, Hoffman et al. (2018, p. 132) find that firms may respond to legislative requirements with a simple risk management approach that avoids addressing the root causes of the problem and does “not tackle major problems related to their business model.” This raises issues of how legislation can encourage and incentivize meaningful corporate responses to mandates. For instance, if corporate liability is based on a corporation failing to adopt adequate HRDD, then regulators or the court system must be able to distinguish adequate from inadequate HRDD. Otherwise, we face the problem of corporations being “focused primarily on documenting a due diligence process to protect itself from blame, while not being primarily concerned that the corporation’s decisions effectively curb business-related human rights abuse...” (Fasterling & Demuijnck, 2013, p. 807).

In the context of their study, Hoffman et al. (2018, p. 116) stated that “the topic of conflict minerals becomes one of supply chain management rather than of individual companies’ legal or compliance divisions alone.” This comment applies to all research on the problem of human rights violations in supply chains. Future research in business ethics can help bring together legal mandates and management practice to improve the impact of our regulatory regimes. We need an understanding of how organizational members understand human rights responsibilities and are encouraged to comply with relevant company policies.

Many corporations struggle with understanding what it means to respect human rights in their situation. The UNGPs, and some mandatory HRDD laws, cover all internationally recognized human rights. Due to the significant number of recognized human rights that could be impacted by business, businesses struggle with determining their responsibilities. McVey et al. (2022) examined how managers within the corporation understood human rights and sought to persuade their colleagues to adopt a rights-based perspective. In some cases, this involved discussing human rights in terms of the financial impact to the company or otherwise reframing the topic in a manner that tones down the communication of the human rights abuse. As the authors argue, “the process of making human rights understandable and manageable can change their form and content.”

HRDD laws must specify the scope of a corporation’s responsibility for human rights violations. Researchers have considered the concepts of “spheres of influence” (Macdonald, 2011) and the different forms of complicity (Wettstein, 2010) in their examination of soft law guidance on this issue. The UNGPs developed new terms—“cause,” “contribute,” and “directly linked”—to determine a corporation’s connection to a negative human rights impact, which then determines the required response. These terms may find their way into mandatory HRDD legislation and they appear in the current draft of the UN BHR treaty. Unfortunately, the UNGPs do not provide clear definitions of those terms. Business ethics scholars can help contribute a real world understanding of the meaning of those terms or suggest alternative approaches.

Finally, as states mandate HRDD through legislation, lawyers will become involved in the compliance process. Business ethics research can help provide an understanding of how that impacts corporate responses. For example, in line with Fasterling and Demuijnck’s (2013) concerns above, some believe that lawyers will push HRDD in the direction of risk management and a tick-box exercise. Thus, the internal governance of HRDD is important. Past research on compliance and ethics programs, and the governance of corporate sustainability efforts generally, provide a useful starting point (Hess, 2021). For example, Radu and Smaili (2021) have examined the impact of corporate governance measures, such as having a board committee focused on CSR and linking CEO compensation to CSR metrics, on improving the company’s social performance.

## Conclusion

The primary goal of this commentary was to use recent developments in the law on business and human rights to show the importance of the integration of law, public policy, and business ethics. Rather than provide an exhaustive look at the issues, it simply used several examples to illustrate how business ethics scholars can provide valuable contributions to understanding the effectiveness of the recent developments in BHR law and propose reforms. Moreover, the hope is to encourage business ethics scholars from any disciplinary background to use their perspectives to help inform law and public policy in their area of interest, including privacy; diversity, equity, and inclusion; artificial intelligence; or any other ethical issue of importance to business.

## Strategy Vs. Ethics?

**Jeffrey S. Harrison**

## Ethics in Strategy

Businesses create a lot of value for stakeholders and society. They provide goods and services, wages for workers, income for investors, and taxes that help to support community infrastructures and a wide variety of government programs and services. Many firms give generously to charities and provide employees time to engage in community programs. This is a short and incomplete list of sources of value provided by businesses. However, in spite of the value they provide, many people have a negative view of businesses and their leaders, believing that they are inherently corrupt and destructive. This perspective is fuelled by widely disseminated information about corporate misdeeds associated with pollution, greed, discrimination, exploitation of workers, bribery, and so forth. In the age of high-speed internet, an abundance of news sources, and social media, businesses are scrutinized more than they have ever been.

These conflicting perspectives about business create a ready forum for ethical debate regarding business strategies. A business strategy is a *discernible pattern of actions* through which a firm attempts to achieve its business objectives or, as strategy pioneer Henry Mintzberg (1978) put it, “a pattern in a stream of decisions” (p. 934). All business strategies have ethical implications because they influence the well-being of stakeholders and society. From a strategy perspective, then, business ethics takes on a practical dimension, exploring the principles and rules through which strategies are formulated and implemented, as well as the outcomes from those strategies on the welfare of a firm’s stakeholders and society at large (Freeman et al., 2010).

Hopefully, a business strategy results in the creation of value for most of a firm’s stakeholders. In the optimal situation, some or all stakeholders receive incremental value from the implementation of a new strategy without reducing value for any stakeholder, a situation known as pareto optimality (Jones et al., 2016). This should be the primary objective of strategic decision makers. However, strategies can also lead to negative outcomes for some stakeholders or for society, a consequence that should not be ignored. Also, the *means* through which strategies are implemented may violate widely held ethical rules or moral codes associated with values such as honesty, fairness, equality, responsibility, justice, or freedom. Firms and their managers should “do what is right.” The fact that there may be disagreement about what this means makes studying strategy from an ethical perspective a fascinating subject. Ethical dilemmas, when managers must choose among options with conflicting benefits and costs, or when the values or moral codes of various parties collide, provide fertile ground for studying business strategy formulation and implementation.

Papers that are submitted to the Strategy and Ethics section, whether conceptual or empirical, should be deliberate in how they fit into the business ethics literature. They should not assume that readers will make the connection. One way to link strategic dimensions to ethical dimensions is to use an ethical theory or framework to build the arguments. Applying utilitarianism, deontology, virtue ethics, social justice, social contracts theory, or a religious philosophy can provide an obvious ethical dimension to a paper. Also, conflicting theories like agency theory vs. stewardship theory *or* shareholder primacy vs. stakeholder theory can enhance arguments about business strategies, although the latter debate has already received so much attention that it might be difficult to make a meaningful incremental contribution to the business ethics literature. Another way to make the connection between strategy and ethics is to draw heavily from the existing business ethics literature, as found in journals such as this one. Simply using a variable that measures corporate social responsibility (CSR) or stakeholder management is not sufficient grounds for inclusion in this section of the journal. An ethics foundation is necessary.

Stakeholder theory has a well-developed connection with strategy formulation and implementation (Bosse & Sutton, 2019; Freeman et al., 2010), and many submissions attempt to use it to give their papers an ethics flavor. However, simply citing Freeman (1984), Jones (1995), or Mitchell et al. (1997) in support of a particular point is not the same as diving into the intricacies of the theory to create well-constructed arguments (Freeman et al., 2010; Harrison et al., 2019). A paper that is built on a stakeholder theory foundation should, at a minimum, describe the actual, potential, or intended consequences for stakeholders and/or society beyond simply demonstrating that a firm is able to make more money. Alternatively, a paper that adopts a stakeholder perspective combined with another ethical perspective is even stronger than a paper that relies solely on stakeholder theory. Remember that stakeholder theory qualifies as an ethics-based theory because it is built on normative concepts associated with relationships between firms and stakeholders such as trust, fairness, integrity, and respect. Purely instrumental arguments that the primary purpose of treating stakeholders well is to make more money are not particularly valuable to business ethics scholarship.

The same ideas apply to corporate social responsibility (CSR). A paper that demonstrates how much money a firm can make if it is a responsible corporate citizen (perhaps with a few moderators thrown into the models) is only going to be attractive if it also explores the ethical ramifications of whatever definition of CSR the author is using. This also means that it is vitally important to clearly define the CSR construct and then, if it is an empirical paper, make sure that whatever measures of CSR are used are consistent with that definition. There are far too many papers in the literature already that make use of a comprehensive measure of CSR that is based on a measure created from data provided by a firm that gathers the data for investors and not for business ethics researchers. In many cases, the construct validity of one of these CSR measures has not been established in the academic research literature. Also, many of these measures are based on hundreds of variables that are processed by the data providers in what amounts to a “black box,” and researchers have to assume that the methods used by the data collection firm are valid.

In papers submitted to the section, examples of moral as well as potentially immoral strategies and actions are welcome. Consequently, also of interest is research on the way corruption, greed, deceit, coercion, and the desire for power influence firm strategies, outcomes, and stakeholder responses. In the next section I will highlight some of the papers that have been published in the journal that fit nicely within the strategy and ethics domain.

### Examples of Published Articles at the Intersection of Strategy and Ethics

Authors who are submitting a paper to the *Journal of Business Ethics* decide which section seems to be the best fit for their submission. In most cases, their preference is granted. Selecting a section may sometimes be difficult because a lot of topics can fit into multiple sections. For example, strategy has potential conceptual overlaps with corporate governance, corporate responsibility, corporate sustainability, global issues, leadership, technology and marketing. This being said, I went through Volume 175 of the journal and found six articles that fit within the intersection of strategy and ethics. Three are highlighted here.

One of the most interesting of the six articles is “Stakeholder Engagement, Knowledge Problems, and Ethical Challenges,” by Mitchell et al. (2022). Stakeholder engagement strategies are of great interest to strategic management scholars and these authors already have well-established reputations in the strategy area. However, the paper is also very strong on the ethics dimension. The authors address several ethical challenges head on in the context of the development and application of genetic modification technologies. Specifically, they examine factors that influence the level to which managers and stakeholders are likely to share a common understanding of either the premises or likely consequences of an action taken by the firm. They not only describe these factors, but they explain how stakeholder engagement can help to overcome problems associated with a lack of common understanding.

Another excellent example is “Losing More than Money: Organizations’ Prosocial Actions Appear Less Authentic When Their Resources are Declining,” by Jago et al. (2022). This article deals with organizational authenticity in the context of prosocial actions. Prosocial behaviors fit nicely into the definition of strategy as a pattern of decisions made by the firm. Furthermore, authenticity is an important ethical dimension related to honesty and trust.

Finally, a third example of a paper that is interesting to both strategy and business ethics scholars is “Competing Logics in the Islamic Funds Industry: A Market Logic Versus a Religious Logic” by Alotaibi et al. (2022). Obviously, this paper fits well within the Finance and Business Ethics section, but because it is about the strategies of investment fund managers, it is appealing also to Strategy and Ethics. Strategic Management scholars are very interested in investment funds and the links between those funds and the investors that support them. The paper qualifies in the ethics area because the strategies are screened against religious philosophy found in Islamic scripture and teachings. Also, the paper explores the influence of Islamic teachings on diversification in these funds. Diversification is an important concept in both finance and strategy.

For the benefit of those interested in the other three articles I identified at the intersection of strategy and ethics, they are: “Building Projects on the Local Communities’ Planet, Studying Organizations’ Care-Giving Approaches,” by Derakhshan (2022); “When Does Corporate Social Responsibility Backfire in Acquisitions? Signal Incongruence and Acquirer Returns,” by Zhang et al. (2022); and “Effect of CSR and Ethical Practices on Sustainable Competitive Performance: A Case of Emerging Markets from Stakeholder Theory Perspective,” by Waheed and Zhang (2022). The range of topics found in these articles, and their unique approaches, reflect the breadth of ideas that can contribute to the intersection of strategy and ethics, and to the broader business ethics literature. Examples of some of the topics and research questions that have a lot of potential to move the strategy and ethics topic forward will now be discussed.

### Future Research on Strategy and Ethics

This is an exciting time to study the intersection of strategy and ethics. So much is happening in the world that has implications for this intersection. Firms need to develop strategies for dealing with changes in the world stemming from new technologies, social movements and upheavals, global health challenges, supply chain issues, and a wealth of other problems that have gained prominence since the start of the new century. As managers deal with these changes, they will need to be sensitive to the ethical ramifications—that is, how their strategies influence stakeholders and society, as well as how they are likely to influence public opinion and, ultimately, their firms’ reputations. In this light, here are a few research questions that seem important to strategy and ethics:

1. How have firm strategies changed in light of social movements associated with diversity and inclusion, and which of these strategies have been the most successful in creating value for firms, their stakeholders, and society? In other words, who are the innovators in this space, and how are their strategies working?
2. How have firms dealt with supply chain shortages, and how have these coping strategies influenced the welfare of employees, customers, suppliers, and communities in which they operate? Have their strategies led to unfair advantages or disadvantages to any particular group? Have supply chain challenges led to unethical behavior?
3. As firms develop or adopt new technologies associated with artificial intelligence, genetic engineering, big data, alternative energy sources, or global value chains, what are the ethical issues that need to be addressed? How are firms dealing with these issues, and which strategies are more or less successful in terms of protecting stakeholder rights and welfare while also enhancing firm performance?
4. What are the strategies that firms use to deal with shocks such as a pandemic or a supply chain disruption caused by a natural disaster (e.g., earthquake, tsunami)? To what extent do these strategies lead to more or less harm to stakeholders and society? Do such shocks lead firms to engage in unethical behaviors or practices? Covid-19 has provided a ready workshop for studying these types of issues.
5. How do institutional, political, and/or societal forces influence the strategies that firms use to deal with shocks such as a pandemic or supply chain disruptions? How do these forces vary globally, and how have different international contexts led to different strategies? To what extent do these forces lead to more or less harm to stakeholders and society, or promote unethical behaviors?

In addition to these types of challenges, the way firms are organizing has changed dramatically in recent years. Global value chains are common in many industries, as are platform organizations (businesses that facilitate transactions across a large number of participants, such as eBay), business groups (i.e., Tata Group), and megacorporations (i.e., Apple, Toyota, and Shell). The increasing popularity of these types of organization have come with a whole range of ethical issues associated with responsibility, fairness, trust, privacy and transparency.

From a strategic perspective, there is already a well-developed literature on the extent to which a firm is responsible for the actions of other actors in its global supply chain (i.e., sweatshops and child labor), and also on how firms cope with cultural differences that promote behaviors such as bribery or lying in their international operations. More research is needed on how a firm’s global value chain strategies influence the welfare of its stakeholders, and whether particular strategies are more prone to ethical dilemmas than others (and how to overcome them without stifling performance). How can firms devise strategies to deal effectively with resistance to globalization (i.e., tariffs, social movements) and how can firms use their strategies to help reduce economic inequality in the nations in which they operate?

Platform organizations and business groups, because their control structures are at least somewhat distributed, offer excellent potential for studying business ethics. The ethical issues associated with these types of structures need to be clearly identified, along with how firms deal with these issues effectively in their policies and strategies. It would be helpful to find out what has worked and what has not, in terms of improving the welfare of and/or harming particular stakeholders. Of course, megacorporations continue to be the subject of ethical debate, and there is still much to do on this topic.

In addition to these research questions that pertain to some of the most important global trends, there are some nagging problems in the strategy and ethics area that have not been adequately addressed:

1. Measurement is a big problem, at least in the CSR and stakeholder areas. For example, Choi and Wang (2009) measured *stakeholder relations* by drawing data from what is called the KLD database, using variables for employee relations, diversity, community relations, the environment, and product dimensions. Then Wang and Choi (2013) used these exact same variables from the same database to measure *corporate social performance*. This is not a criticism of the authors, but rather an example of a huge problem in the field—both of these papers passed through a rigorous review process at a top management journal. Moving forward, we need to make sure that, once we have clearly defined our constructs, we also make sure that there is consistency between the construct and the way it is measured. This principle applies not only to the CSR subject area, but to other topics in business ethics.
2. Beyond the big commercial databases whose primary source of income is investors, there is very little publicly available data that researchers can use to examine the value firms create or destroy for stakeholders (e.g., stakeholder welfare). Given that so many of the interesting topics in strategy and ethics pertain to this type of welfare, creation of new databases or even a consistent set of data collection tools would be very beneficial in advancing knowledge in the field.
3. The most common dependent variable in empirical studies in strategic management is financial return in the form of profit, shareholder returns or something similar. While profits are important to the ability of a firm to achieve its objectives, including the creation of value for stakeholders, there are infinite possibilities for new studies in this section if researchers spend more of their efforts examining other dependent variables. CSR is sometimes used as a dependent variable, and this is a good step (as long as the construct definition and measures are consistent). Other dependent variables could include community welfare, employee welfare, supplier welfare or customer welfare, just for a start. Some of the big databases that are already in use contain data that may be useful in creating these types of measures. Qualitative methodologies and methods can also be very helpful. The key is to think about the stakeholder and societal value that is created or destroyed as firms implement their strategies (Harrison & Wicks, 2021).
4. Finally, new theory is sorely needed. Among the most used theories at the intersection of strategy and ethics are stakeholder theory, agency theory, stewardship theory, or salience theory. Scholars also borrow theory from other disciplines to support their arguments, which is good, but where are the new theories that are specific to strategy and ethics?

In summary, the intersection of strategy and ethics offers many opportunities to address some of the problems associated with global changes and challenges. In addition, there are research opportunities for advancing the field through cleaning up some of the remaining conceptual and methodological problems that have impeded research in the area for many years. Of course, the overarching aim of the journal is to develop more of an awareness of ethical issues in business and ways that businesses can become more ethical, and do more good in the world, thus reducing the propensity for people to look at business as clashing with ethical principles—ethical businesses strategies instead of “strategy vs. ethics.”

## Toward a Framework to Explore Unethical Business Models

**Sheila Killian**

## Introduction

This essay proposes a framework for research that critiques not only the ethical implications of business practice, but also of business models. If there are embedded ethics, there can also be embedded unethicality. If there are social enterprises, surely there are also antisocial ones (a categorization as yet undefined), who by their very business model, promote unsustainability or exacerbate inequality. Beyond the idea of bad apples or even bad barrels, which has been well explored in this journal (the latter implying a flaw in a barrel designed to be “good”) there are business structures hiding in plain sight which by their very design embody and reify unethical practice.

The negative impact of some businesses is clear, particularly if the model is one that operates outside the law. With the proviso that laws vary more widely by jurisdiction than ethics do, and may be subject to political influence, or other cultural factors, illegal activities are often, although by no means always, unethical. The sale of heroin, for instance, and armed robbery are both for-profit enterprises which exist outside the boundaries of legality and therefore of respectability. This makes research on the ethics inherent in their models appear redundant, a waste of scholars’ energy and resources. It appears obvious that these models are unethical because we first see them as illegal. But in fact it is the other way around. The reason that these things are illegal, the trigger for their being sanctioned by law, is that they have been deemed at a particular place and time to be unethical and harmful. Correspondingly, the legalization of formerly criminalized activities reflects a changing understanding of how society should operate. Law is a *response to* societal understandings of ethics. Therefore, while it offers one option to reveal the kinds of models we, as scholars, might address, it does so at a delay, lagging societal awareness.

Consider activities that have recently been banned. The sale and deployment of cluster bombs, for example, was outlawed in 2010 by the Convention on Cluster Munitions (Oslo Convention) due to their indiscriminate and harmful nature. Prior to that date, they were still harmful, but the law would not have acted as a flag to this effect. Right now, their illegality points toward their unethicality. It is our role as researchers in this field to question business models that are legal, but which have built-in unethicality which may 1 day be banned, or which might deserve such sanction even if it is politically untenable. At the time of writing in early 2022, proposals are under consideration in New Zealand which will effectively, over time, outlaw the sale of cigarettes. Why is the New Zealand government taking this position? Because tobacco is inherently harmful, killing half of its users, according to the World Health Organization. Unlike alcohol, that is harmful with over-consumption, the World Health Organization observes that “there is no safe level of exposure to tobacco” (WHO, 2021). The business model for supply of tobacco depends on knowingly selling a product that will harm consumers. It is not that some, or even many, tobacco companies practice unethical behavior. It is the model, the design, that is inherently unethical.

Like tobacco, we know there are other industries, sectors and companies with business models which are (currently) legal yet ripe for analysis, containing elements that are inherently, almost by design, damaging or unsustainable. Can we frame this in terms of ethics, and what would such an approach tell us about business ethics itself? By default, scholars approach ethics in relation to decisions, and when we apply this to business, our perspective can tend toward a micro rather than a macro view. As well as looking at bad practice, bad apples, and bad barrels, the ethicality of business models would be an interesting topic to explore further in this journal.

A desire to do so, as a community of scholars, begs the next question: how do we identify such businesses? Harrison and Wicks (2021) have written on this topic in the context of stakeholder theory, providing a basis for identifying strategies that might be considered unethical by stakeholders and exploring the response of firms. A complementary approach would be to develop a framework. What we are seeking to identify is not unethical practice on an individual firm basis, or even unethical practice that is widespread but avoidable within particular industries, such as low wages in hospitality, but business models that depend on specific unethical elements. It is common, for instance, for food delivery workers to be poorly paid, but, arguably, the business model does not depend on those low wages. A delivery service could exist which charged more and paid well. However, other business models depend clearly on some harmful or unethical dimension. An example might be the tobacco industry, as noted above, or the provision of essay-cheating services to students. The purpose of this commentary is to explore a framework to identify and examine these.

### Toward a Framework of Unethical Business Models

An approach to the question of how to identify unethical business models can be made either deductively or inductively. We might, for instance, locate a pre-existing code that can be used to classify them, or we could inductively explore businesses with significant ethical issues, and attempt to isolate the specific elements on which they rely. Using a code, the law, as we have seen, is somewhat useful ex-post, but overall it is inadequate to the task, being slow to respond, subjective in some ways, insufficient and geographically inconsistent. Another potentially useful rubric might be the criteria applied by ethical investment funds. The ethics of ethical investing have long been scrutinized in this journal,^1^ and as the field transitions into ESG metrics and socially responsible investment factors, major issues that emerge are a lack of transparency, and a lack of convergence across the field on how and which criteria should be applied (Widyawati, 2020). For these and other reasons, this essay adopts the second of the two possible approaches flagged above, and explores in an inductive way business models that appear fundamentally harmful or unethical, aiming to synthesize a tentative framework for evaluation. Note that the dimensions explored may not, on an individual basis, brand a business model as unethical. In aggregate, however, they may provide a frame by which such business categories could be considered.

The tobacco industry may be seen as an example of a business that causes inherent harm, in its case primarily to the human health of consumers. Even where the tobacco is grown organically and sold on a fair trade basis, with well-remunerated workers and a supply chain free of exploitation, the sale of the product itself is always, even at low levels of consumption, damaging. Inherent harm, as a dimension of unethical business models, could also apply beyond consumers. Some forms of mineral exploration, for instance, may cause irreparable harm to the environment. A business that requires such approaches, especially if the exploration is not strictly necessary, may be given a high score on the dimension of harm.

Another dimension may be dependence. Some for-profit models are built on the foundation of locking consumers or suppliers in to future purchases. For example, newer Apple products which do not have a standard headphone port require users to either buy an adaptor, or to depend on the company to supply such accessories. A related element is built-in redundancy, or a lack of backwards compatibility. In gaming, for instance, some new games require upgrading of the hardware needed to play them, and in some cases the newer consoles will not support older games. Another example is phone batteries designed to degrade after a certain length of time, or digital cameras which become redundant even while functional when there is no longer software available to link to newer computer operating systems. Practices of creating dependency create moral hazard for the design of entire business models built on this behavior.

Another element is facilitating or encouraging unethical behavior on the part of others. Essay-writing services for students are an example, or those parts of the dark web that depend on platforming racist groups. A fourth dimension might be appropriation: privatization or theft of common resources like water supplies, radical overfishing, the patenting of genetic material, or, spectacularly, the February 2020 call by the Adam Smith Institute for the privatization of the moon. Deception is also a clearly unethical practice, and some businesses, notably in the field of natural medicine, beauty or diet products, promise far beyond what they can or do deliver. A congruent idea is that of an industry creating a problem for which they are the only solution, as in the case of some “smart food products” or much of the beauty industry.

Another dimension, not unconnected but distinct, is that of predation, or predatory behavior. An example is the selling to vulnerable groups of payday loans which only become profitable if the borrower does not pay on time. The loans are marketed in a disingenuous way, based on early repayment, but the business model depends on default. More indirectly, predation might include the sale of products that promote unrealistic beauty standards to young women in particular.

Exploitation is intrinsic to business models ranging from the use of near-forced labor to parts of the gig economy, or creative outlets that do not pay performers a living wage. Even the language used in the creative industry is revealing: artistic work is now routinely called content, relegating it to something that serves the distribution channels, rather than the other way around.

### A Stylized Framework

**Fig. 1:**
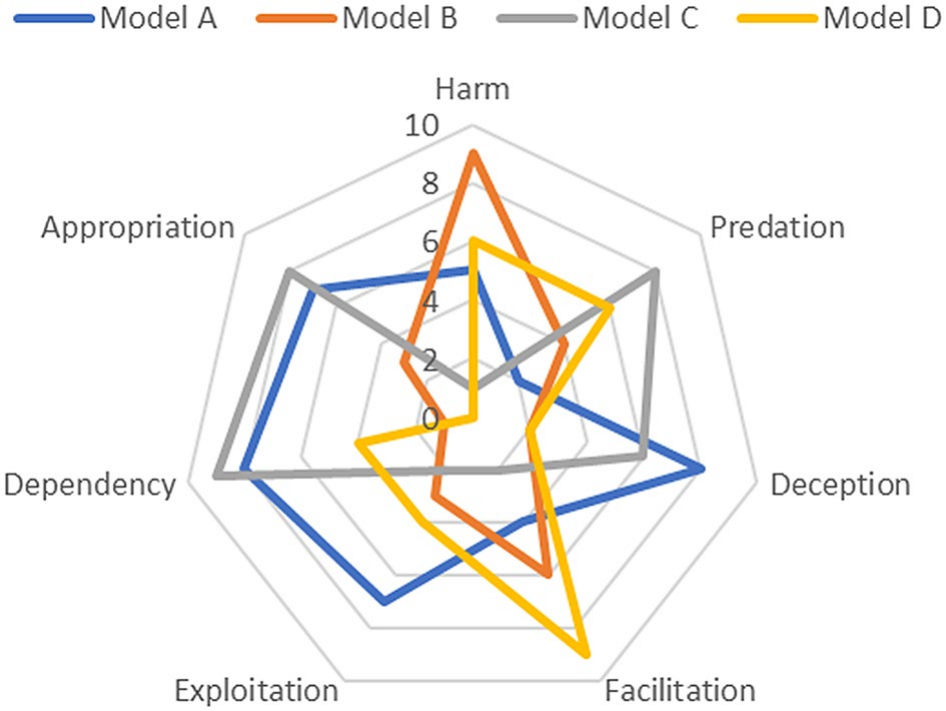
Framework mapping the ethicality of business models

These seven dimensions, harm, dependence, facilitation, appropriation, deception, predation and exploitation, may act in overlapping ways to support a business model with an unethical core. Such a simple framework would allow researchers to map the dependence of any given business model on each of these dimensions, perhaps with results as shown graphically as in Fig. 1, below, for four stylized, pro-forma business models labeled A to D.

This initial framework requires refinement in three key ways, and research is invited to explore correlations and redundancies, to add missing elements and dimensions, and, critically, to devise a means of scoring, perhaps based on large datasets.

### The Means of Distribution

In applying the framework to business, it may be important to once again step back from examining products and services, to focus on elements of distribution. A central tenet of Marxism, eloquently expressed in W. E. B. DuBois’s posthumous autobiography (1968) was: “for working people to be free, they must *seize control of the means of production*.” He was thinking of slavery and forced labor in the cotton fields of the US South, where the invention of the cotton gin had made free labor in plantation fields extremely profitable for the few, but back-breaking and imprisoning for the many. In that pre-globalization context, he correctly saw the planation system as a generator of inequality, and a perpetuator of poverty and slavery. If the people were to be free, control of this means of production needed to be in wider hands. Production was the controlling element of its time. If you looked and asked: “Who are the wealthy? And what have they that the poor do not?” the answer would be factories, mills, mines, farms.

Our current system of financial accounting developed in the service of this old model of capitalism, accounting to the providers of capital on how their investment has been deployed in order to *produce* something. We assess companies—“account” for their activity in a literal sense—by evaluating the cost of the goods they produce and the revenue they obtain by selling them. Financial accounting developed to account for a commodity-based business—sales, cost of goods sold. Services are accounted for using the same structure, and thus are seen, in a way, as a special class of goods. Through a financial accounting lens, a service company is seen as selling something that is ultimately consumed in the same way as, say, a sandwich, or a widget. Profit is accounted for by taking the revenue from “selling” the service, and subtracting in some sense the cost of this sale. This fundamental idea—that the value generated within companies is linked to what they *produce—*has shaped our thinking about how business works. It colors how we theorize on how invested capital will be concentrated or distributed or generate value within or outside an organization. It forms the basis of financial accounting and delineates how we think about business ethics and about power, capital, production, value, and inequality. But is there a new element to consider?

Consider *The Evergiven*. In March of 2021, the unfortunate captain of the container ship, *The Evergiven* ran aground near the village of Manshiyet Rugola, blocking the Suez Canal. For a week, the world watched efforts to free it on nightly news broadcasts, daily newspapers, and in real time on social media. It was just one ship, run aground in a place most people had never heard of. But it mattered, somehow, to us all. Factories in countries dominated by production open and close all the time, and those of us in the privileged countries dominated by consumption barely notice. But let a ship block up the Suez Canal for a week, and we sit up, our fingers poised over the “Buy Now” button, wondering how this disruption to shipping might affect us, how it might affect our spending.

We now live in a globalized age of networked capitalism and consumerism, where rising inequality and its associated societal damage is driven not just by a few individuals controlling the means of production of valuable goods or services, but by distribution. Amazon destroys millions of items of unsold stock every year. Consider the business model that makes this more profitable for Amazon than selling the items cheaply. They have found a way to make money from the offer of distribution, even if no goods are sold or consumed. When billionaire artists like Beyoncé and JayZ set up Tidal in competition with Spotify, it was clear that, even for superstar producers of music, the money lies in distribution.

More generally, on a basic level, failures of distribution impact disproportionately on the poor. Wealthier people can stockpile a little, or time their spending. Those without a financial buffer are more reliant on getting that fill of fuel when they need and can afford it—they can’t afford to wait. For a starker example, consider the distribution of vaccines in the Covid-19 pandemic, booster vaccines were rolled out widely in countries with good health infrastructure, while many in the global South were still waiting for their first dose, putting their health and their lives at risk.

Perhaps our system of financial accounting, which developed to provide an account to the providers of capital about their commodity-based businesses, is no longer appropriate to this new era. Perhaps it even serves to occlude the means by which an elite can generate private profits without producing any goods. Perhaps we need to reconsider what it means to run a successful business, what it means to be productive in a real sense. Amazon does not need to provide a commodity the world needs, and price it accordingly, to be profitable. It gives us something else we appear to desperately want: instant consumption of everything, 24 h a day, delivered to our homes “for free.” Their model does not need to know or care about the utility of the goods. It simply needs us to want to buy something, anything at all, as the control the channels for an ever-widening range of goods. What they are selling us is consumption itself. The only desire they need to awaken is the desire to purchase. Once that is established, profits accrue to them, the distributor, regardless of whether they accrue to the producers. To an extent, then, they may be facilitating this harmful practice of over-consumption; their model may be predatory in relation to smaller, local suppliers; it may be designed to create dependency—not absolutely, but to some extent that could be scored using the framework above. Applying the framework with a distribution as well as a production lens could therefore be useful.

## Conclusion

Of course, there are still production-based problems. Sweatshops persist. The ILO (2022) estimates about 40 million people are trapped in forced labor, a quarter of whom are children.^2^ Neither is it a choice between the merits of production or distribution. Modern modes of production are themselves complex and networked, dependent on interwoven pathways of materials and processes that span the globe. Distribution underpins production. And if we stand and look around, as W. E. B. Dubois may have done, and ask: “Who are the wealthy? What do they have that the poor do not?” we will find ourselves looking not at the finery of plantation-owners, but at billionaires taking daytrips to space whose wealth is founded not on production, but on the distribution of goods, and the curation and channeling of our ideas and even of our relationships, thereby creating a lasting dependency.

This proposed framework, while imperfect, can be progressed to help identify unethical business models. Having done so, many aspects of the business would merit examination: design and packaging of the product or service; marketing strategies; price points and availability; the CSR strategies of companies operating this business model; social impact accounting. Researchers could explore the ethics of people who work in, patronize or invest in such businesses, with due regard to issues of power, choice and voice. The framework could also be applied to aspects of otherwise benign industries, such as education or healthcare. Closer to home, for example, it could be applied to the model of academic publishing, considering how young scholars need to corral their ideas into narrow channels that will reach publication in the few outlets that will be recognized by their employers. There are design issues to be explored in terms of the cost of open access, the dominance of English language, the pressure placed on our future scholars by tight and demanding tenure tracks, the role of encouragers and gatekeepers, the motivations created by our promotion and recruitment systems.

Overall, this approach could complement work that seeks out examples of good or bad business ethics, by examining business systems, their morality and who they serve.

## Taboo and Totems in the Study of Social Ventures, Entrepreneurship, and Small Businesses

**M. Tina Dacin and Julia Roloff**

## Introduction

Descriptions of entrepreneurial activities range from glorifying entrepreneurs for their willingness to take risks, hard work, and innovativeness to raising general suspicion that entrepreneurs cut corners to get their business going and are motivated by self-serving goals. The literature on social enterprises, family businesses, start-ups, and small- and medium-sized enterprises (SMEs) often belongs to one extreme or the other: either entrepreneurs or their ventures are studied as exemplars for outstanding achievement and contributions to society (e.g., Fowler et al., 2019), or they are studied for their deviance. Studies that present small businesses, family firms, and social ventures as places where well-intentioned people struggle to walk the talk about responsible and sustainable business practices are rare. However, without research that captures both the good and the bad, we fail to create management theory and recommendations that are relevant to business practice.

We observe contributions to the literature on social entrepreneurship and small business in which successful business ventures and their leaders are revered in a way that reminds us of totems. These examples are adverted to as proof that socially and environmentally responsible business ventures can also be financially successful. This idea is essentially so precious that criticizing these ventures, their operations or their leaders essentially becomes taboo. We explore the notions of totem and taboo and how they dominate our thinking and push for a move toward a more nuanced and balanced approach to research in this domain.

The terms totem and taboo have a long history in anthropology and anthropologists have described practices of totemism among ethnic groups from various continents. For example, Frazer (1887, p. 1) wrote: “A totem is a class of material objects which a savage regards with superstitious respect, believing that there exists between him and every member of the class an intimate and altogether special relation.” A wide range of cultural practices is described in connection with totemism, ranging from worshipping the totem to identification with it. Totemism comes with taboos, such as the taboo against killing or eating any individual of the totem species since this would be considered cannibalism. Freud (2013/1913) claims in his book “Totem and Taboo” that this identification with a totem is the social institution that laid the ground for an incest taboo, as two people who identify with the same totem will not “consume” each other sexually. According to Freud (2013/1913), the origin of taboos is unclear, as he believes them to be older than religious and moral prohibitions and they come with no clear explanation. He borrowed largely from the work of writers such as Van Gennep (1904), who referred to a taboo largely as a prohibition that could be grounded in religious, moral, or even social foundations. While taboos and their associated prohibitions can be behavioral, they can also be conversational—neither talked about nor publicly debated and discussed (Sabri et al., 2010).

We assert that some contributions to the business ethics literature show signs of totemism and of taboos that lack justification. Sabri et al. (2010) suggest that taboos are “cultural productions” that are crafted and embedded in social and historical contexts. The accelerated evolution of social ventures as a panacea for society’s ailing aid and impact sector has imposed enormous pressure to present only the good while downplaying the bad sides of these ventures. We see case studies that do not dare criticize any aspect of a social venture, or those that present claims of successful leaders as gospel without critical distancing or considering the possibility that an exemplary venture may also be fraught with weaknesses. Here, social ventures are approached as totems. Taboos play a role when entrepreneurs are studied for their deviant behavior, such as their propensity to lie, distort and/or misrepresent information in order to gain support from investors and clients, but there is neglection of discussion of those who do not resort to such behaviors (e.g., Ji et al., 2019): in this case, the idea that entrepreneurs forego a business opportunity because of their individual or organizational values is the taboo. This tendency of researchers to revere totems and observe taboos has led to a number of problematic outcomes that limit our ability to meaningfully unpack this literature. We encourage researchers to consider how their theoretical framing and methodological approaches can introduce or remove bias in their research.

### Bias Introduced by Theoretical Framing

First, for the most part, the social venture and small business literature tends to focus on hybrid forms of organizing, where it is assumed at the outset that there are tensions across the logics (most often social and commercial) which are blended to guide and govern the enterprise. The assumption is that the logics driving social ventures compete and are largely incompatible. However, this assumption may be unfounded and ignores the fact that firms generally operate in pluralistic institutional spheres that involve the simultaneous juggling of multiple logics. An example is a family firm, which has to balance the needs of the business, the family and those of the firm’s stakeholders (Signori & Fassin, 2021). We do not want to discount the rise of conflicts or tensions because of multiple logics. However, an implicit assumption that there is incompatibility from the outset is largely misguided.

Second, there is a romanticized narrative that often accompanies small, entrepreneurial enterprises and family businesses, and especially in the discourse around social ventures. Indeed, most of the narratives describe the work of social entrepreneurs in a positive light. Social entrepreneurs are heroic totems who seek and gather accolades at a variety of field-configuring events led by various foundations and luminaries. At these events, entrepreneurs are highly celebrated, reinforcing a cycle of pointing out the positive in an extreme form of hero worship. These same heroes go on to enjoy a cycle of success receiving awards from one event to the next. As they continue their momentum of success, these heroic social entrepreneurs are rarely scrutinized, and in fact their stories are careful curations of their origin, work, and impact. In so doing, we know of little to nothing in published scholarly work about the darker side of these actors and their ventures, although recent examples in the news media shed light on some controversial activities (Waldie, 2020).

Third, by focusing on totems and ignoring more mid-range dialogue about the positive and darker sides of entrepreneurial activity, we privilege the role of individual actors and ignore the collective side of entrepreneuring (Montgomery et al., 2012). In fact, many social ventures involve groups of individuals. Collective sets of actors such as key stakeholders, including partners, funders, and community actors, should not be discounted nor ignored. In fact, in community settings, much good comes from the efforts of collective social innovation (Dacin & Dacin, 2019).

Taboos can influence researchers when they develop their theoretical frameworks. For example, two recent studies investigating how entrepreneurs describe their ventures on crowdfunding platforms exemplify this problem. Defazio et al. (2021) study the impact of prosocial framing in these descriptions, while Calic et al. (2021) investigate whether using Machiavellian rhetoric makes projects more or less successful in receiving financial backing. Both studies address the same research question on how project descriptions influence funding success and failure, but they approach this question from diametrically opposed positions, assuming that investors are either drawn to socially impactful projects or manipulated by ingratiation and stories of betrayal. Together, these two studies provide a more complex understanding of venture presentations on crowdsourcing websites. However, it is often feasible to design studies that capture a wide range of motivations and actions and therefore allow exploration of both the good and the bad in the same study.

In particular, research on social ventures has been limited by the tendency and practice to establish totems and to avoid taboos. To what extent can we be confident that the literature to date has rendered valuable knowledge and insight about whether social ventures have produced lasting, systemic, and structural change in the range of complex, inter-related problems, such as homelessness and poverty? By assuming that social ventures are inherently good, there is a tendency to avoid comparison to other forms of doing good, take impact as given, and often inspection is seen as harsh and also is often unwanted. The mythical status attributed to social ventures being “inherently good” means we tend to overlook potentially viable alternative approaches such as public, private, and charitable organizations, cross-sector partnerships and corporate philanthropy (Dacin et al., 2011). In fact, in the last two decades other pathways for good may have been subjugated to the allure of the social enterprise over other alternatives.

### Bias Introduced by the Methodological Approach

We want to encourage researchers to be mindful of tendencies toward glorification and vilification in the research of social ventures, entrepreneurship, and small businesses. There are examples for studies that are designed to identify virtuous as well as problematic patterns.

Such research can be motivated by unexpected observations such as the study by Dorado et al. (2022) of a successful social bank that struggles with employee retention. Dorado et al. investigate the reasons why this value-driven social enterprise sees so many of its employees leaving after a few years of service. A similar inquiry—but based on large population samples—is conducted by Brieger et al. (2021), who study how social value creation, meaningful work and burnout are related. This research helps us to understand the price people pay when working for a social venture as they perceive pressure to set private needs and goals aside.

On the other hand, keeping a business on the right path is challenging to do. For example, Sendlhofer (2020) describes how employees of a SME that is recognized as a corporate social responsibly (CSR) pioneer started decoupling from the firm’s praised practices. Moreover, they procrastinated when it came to creating new socially responsible practices because their past success made them confident that their firm would continue to excel anyway. Similar observations of mission drift are made in the microfinance sector when over time lenders tend to prefer more capable clients over more needy ones (Beisland et al., 2019). These studies help us to understand the messy nature of business ventures aimed at creating valuable outcomes for society and highlight that these organizations can hardly be categorized as good or bad, but that they are operating between high aspiration and very real human and organizational limitations. Limitations and aspirations are best studied with research questions that are interested in a wide range of observations.

Other examples of studies avoiding bias can be found in the research on what motivates entrepreneurs that capture responses ranging from egoistic to altruistic motivations. For example, a study on indigenous entrepreneurs demonstrates that emancipation through entrepreneurship can take on many meanings, as some entrepreneurs work toward making themselves autonomous while others want to instigate change in their community (Pergelova et al., 2021). If the researchers had only asked questions on how indigenous entrepreneurs seek to help their community, we would have learned less about their need for individual autonomy. Another study avoiding taboos investigated the role of SME in peacekeeping was able to capture dynamics that promote peace and others which foster conflict (Joseph et al., 2021): this study highlights that creating income and employment in a conflict-ridden community is not sufficient to promote peace—in order to make a contribution, firms needed to reduce inter-group conflict between Lebanese citizens and Syrian refugees within the firm. If the researchers had only been interested in observing how firms contribute to peacekeeping, they might have failed to observe that in some firms conflictual beliefs are perpetuated. Such findings are feasible since the studies were designed to capture motivations, beliefs, and behaviors along a continuum between virtuous and beneficial, on one side, and deviant and harmful, on the other. Thus, we can learn more about the struggles people experience when they are trying to do the right thing, create ventures that produce more than financial value and work toward keeping these ventures on the right path in changing environments.

These studies link observations on the individual, organizational and societal level, such as exhaustion and decoupling among employees and unintended problematic consequences of entrepreneurial activities at organizational and community level. By conducting research that sheds light on the gray zones of entrepreneurship and managing small ventures, we can create theories that address problems that entrepreneurs and their teams experience every day. A focus on only one end of the continuum between virtuosity and deviance blinds us to the nuances and complexity of entrepreneurial activities and the difficulties that people experience when they are trying to “walk the talk” and not to lose their path as they continue walking.

### The Path Forward

To reduce our focus on totems and taboos from research on social ventures, family businesses, start-ups, and SMEs, we need to be open to observing the good and the bad in any empirical context, and we need to find ways to describe and explain a complex and embedded social system, in which people with good intentions fail to deliver desired outcomes and people with questionable intentions create value for others. Only by designing studies that can capture the complexity of doing business in a dynamic environment can we create theory that provides an accurate description of our messy reality and permits us to develop solutions that work under given constraints.

We invite efforts for more systematic research on the motivation and rationale as to why some businesses work toward more responsible and sustainable business practices and others neglect to do the same. Future research ought to inquire whether good, moderate, and poor practices can be explained by factors influencing enterprises at a societal or cultural level, such as institutional and legislative pressures, market demands, or the absence of such factors. Do inadequate organizational resources, capabilities, or resistance to change explain why businesses fail to do better? Are individual factors such as motivation, values, and beliefs the reason why entrepreneurs, managers, and employees engage with or decouple from such goals? What is the role of stakeholders and partners in encouraging or discouraging responsible business practices? How can we better understand the unintended negative consequences of doing good?

Moreover, we are also interested in a critical analysis of our research traditions. To what extent do the totems we perpetuate maintain the façade or serve as a mask for material or ideological interests? Where does the dark side reside? Is it hidden, just below the surface, or more deeply embedded in the core of entrepreneurial activity? Moving forward, what can we learn about social impact creation by focussing more on how ventures capture value instead on how they create it (Bacq & Eddleston, 2018)? How do various pathways to social impact compare? Are social ventures more effective than other forms of impact such as cross-sector partnerships (Vurro et al., 2010)?

We are looking forward to a discourse on social ventures, family businesses, start-ups, and SMEs that is ready to question old assumptions and ready to capture the potential but also the limitations of individuals and collectives to create socially beneficial ventures.

## Footnotes

1 See, for instance, Schwartz (2003) and the considerable body of work citing that paper, or, more recently, Widyawati (2020).

2 See https://www.ilo.org/global/topics/forced-labour/lang--en/index.htm for estimates of the level of forced and child labor worldwide.
